# Impact of the Great East Japan Earthquake on feeding methods and newborn growth at 1 month postpartum: results from the Fukushima Health Management Survey

**DOI:** 10.1007/s00411-016-0636-7

**Published:** 2016-02-13

**Authors:** Hyo Kyozuka, Shun Yasuda, Makoto Kawamura, Yasuhisa Nomura, Keiya Fujimori, Aya Goto, Seiji Yasumura, Masafumi Abe

**Affiliations:** Department of Obstetrics and Gynecology, School of Medicine, Fukushima Medical University, 1-Hikarigaoka, Fukushima, 960-1295 Japan; Radiation Medical Science Center for the Fukushima Health Management Survey, Fukushima, Japan; Department of Public Health, School of Medicine, Fukushima Medical University, Fukushima, Japan

**Keywords:** Natural disasters, Environmental disasters, Pregnancy, Maternal stress, Breastfeeding, Formula feeding, Growth status

## Abstract

This study examined the effects of three disasters (the Great East Japan Earthquake of March 11, 2011, followed by a tsunami and the Fukushima Daiichi Nuclear Power Plant accident) on feeding methods and growth in infants born after the disasters. Using results from the Fukushima Health Management Survey, Soso District (the affected area where the damaged nuclear power plant is located) and Aizu District (a less-affected area located farthest from the plant) were compared. In this study, newborn and maternal background characteristics were examined, as well as feeding methods, and other factors for newborn growth at the first postpartum examination for 1706 newborns born after the disaster in the affected (*n* = 836) and less-affected (*n* = 870) areas. Postpartum examinations took place 1 month after birth. Feeding method trends were examined, and multivariate regression analyses were used to investigate effects on newborn mass gain. There were no significant differences in background characteristics among newborns in these areas. When birth dates were divided into four periods to assess trends, no significant change in the exclusive breastfeeding rate was found, while the exclusive formula-feeding rate was significantly different across time periods in the affected area (*p* = 0.02). Multivariate analyses revealed no significant independent associations of maternal depression and change in medical facilities (possible disaster effects) with other newborn growth factors in either area. No area differences in newborn growth at the first postpartum examination or in exclusive breastfeeding rates were found during any period. Exclusive formula-feeding rates varied across time periods in the affected, but not in the less-affected area. It is concluded that effective guidance to promote breast-feeding and prevent exclusive use of formula is important for women in post-disaster circumstances.

## Introduction

**Fig. 1:**
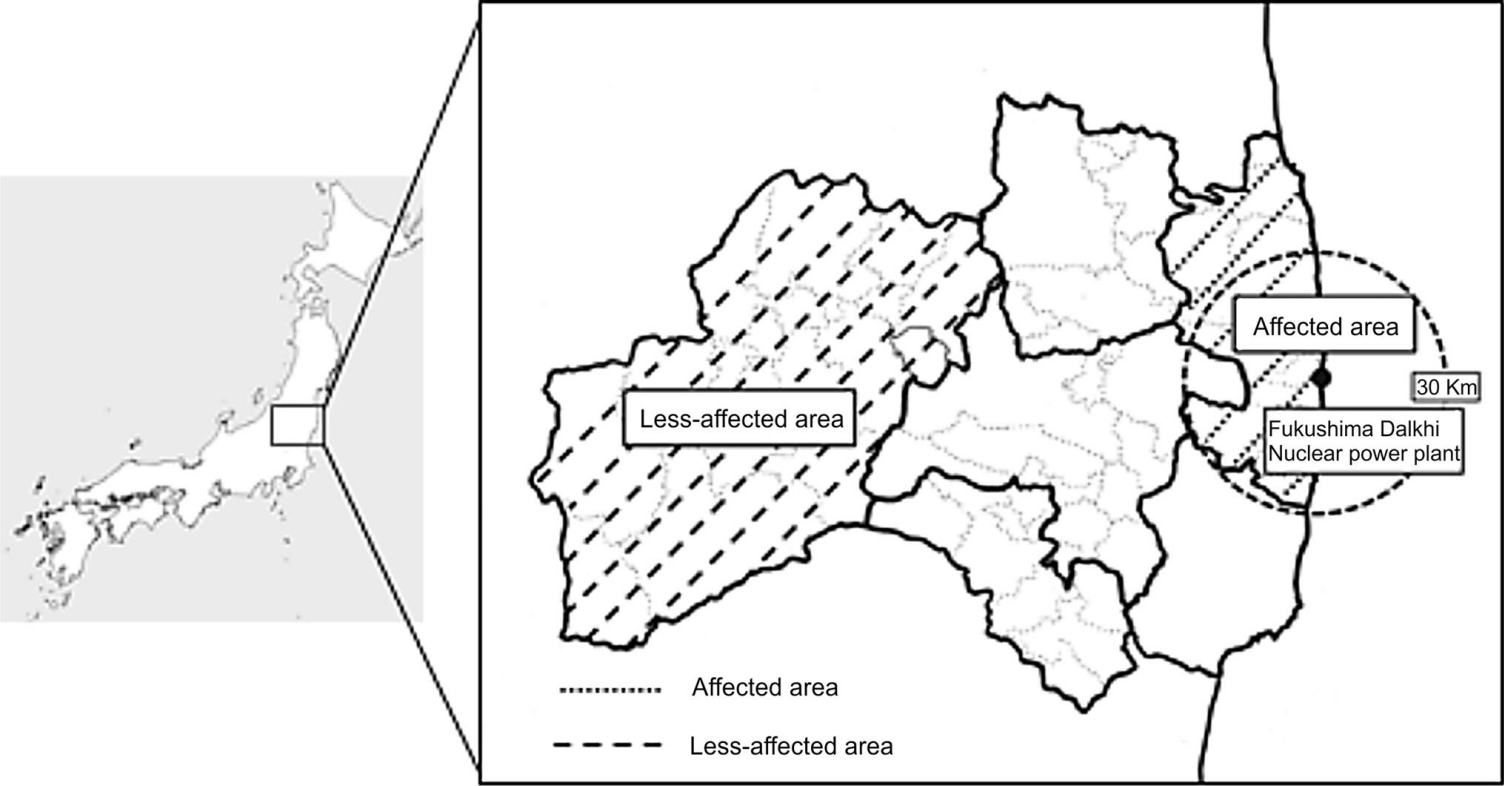
Map of Fukushima Prefecture and affected areas. The affected area includes Soso District, and the less-affected area includes Aizu and Minami-Aizu districts

The Great East Japan Earthquake of March 11, 2011, followed by a tsunami, and the subsequent nuclear accident at Fukushima Daiichi Nuclear Power Plant have been the most catastrophic events in recent Japanese history. The tsunami caused thousands of deaths and devastated whole communities leading to mass evacuations in Fukushima Prefecture. In particular, many people (including pregnant women) in Soso District, on the coast of Fukushima Prefecture, were forced to evacuate immediately because the earthquake and tsunami had caused direct damage to their houses. Further, the government designated many regions of Soso District as falling within the evacuation zone after the nuclear disaster (Fig. 1).

A previous study on the effects of the Great East Japan Earthquake and the subsequent nuclear disaster on pregnancy outcomes reported no significant adverse outcomes across Fukushima Prefecture (Fujimori et al. 2014). Nevertheless, several studies have demonstrated the aggravation of different forms of malnutrition during and after disasters and emergencies (World Health Organization 2000; Singh 2010). Evidence suggests that the methods of feeding infants during the 2 or 3 years following birth can affect subsequent child development (Liu et al. 2000; Richard and Martin 2008). Accordingly, proper feeding and growth in the early stages following birth are of major importance in public health.

Breastfeeding is advised during natural disaster situations by the World Health Organization (WHO) and UNICEF (2003) due to the many health benefits that breast milk provides. However, a decline in breastfeeding rates is noted during emergencies, with a corresponding increase in formula feeding (Adhisivam et al. 2006). In developed countries, formula use is an option for infant feeding due to easy access to clean water, electricity, and medical care. However, natural and environmental disasters can disrupt water and electricity supplies, leading to circumstances that make formula feeding potentially dangerous due to polluted environments (Gribble and Berry 2011; Sulaiman et al. 2015). Additionally, research has shown that misconceptions about alleged unhealthy effects of breastfeeding during disasters can lead to uncontrolled and poorly targeted donations of infant formula (Gribble 2013). This has the potential to increase infant mortality if donations encourage mothers to replace breastfeeding with formula in areas with contaminated water [United Nations High Commissioner for Refugees (UNHCR) 2009].

To the best of our knowledge, there are no studies that have specifically investigated feeding methods and growth in newborns in large-scale post-disaster scenarios, such as those observed after the Great East Japan Earthquake. In this study, therefore, the Pregnancy and Birth Survey from the Fukushima Health Management Survey (Yasumura et al. 2012) was used to investigate the effects of the Great East Japan Earthquake, tsunami, and the subsequent nuclear disaster on the feeding methods and growth in the infants born following the disaster. Disaster effects were evaluated by comparing newborns of mothers who lived in an affected area with those born to mothers who lived in a less-affected area.

## Materials and methods

### Study design and sampling method

In the present study, results of a maternal survey questionnaire that was part of the Fukushima Health Management Survey (FHMS) were analyzed. The methods of the FHMS and Maternal Survey have been described in detail previously (Yasumura et al. 2012). Briefly, the maternal survey was a population-based study conducted in Fukushima Prefecture as part of the FHMS to assess the health conditions of pregnant women and newborns affected by the Fukushima Daiichi nuclear disaster. The questionnaire addresses antenatal health, delivery method, maternal mental health, and the results of the 1-month child health checkup. The survey divided Fukushima Prefecture into seven districts and involved pregnant women who received maternal and child health handbooks between August 1, 2010, and July 31, 2011, as well as their newborns. These handbooks help maintain a record of women’s antenatal and postnatal checkups by physicians. Questionnaires were sent by mail from January 18, 2012, and the mothers were instructed to return the surveys within the following 2 weeks. Mothers were also asked to refer to their maternal and child health handbooks when completing the questionnaire to help with recall.

For the present study, the coastal Soso District of Fukushima Prefecture was defined as an affected area. Soso District was directly damaged by the earthquake and tsunami and is located closest to the nuclear power plant (Fukunaga and Kumakawa 2015). Aizu and Minami-Aizu districts, which are located in the mountainous inland of Fukushima Prefecture, were combined for the present study and defined as a less-affected area. The combined area sustained relatively little damage and is located—compared with the other districts of Fukushima Prefecture—farthest from the nuclear power plant.

Twins and newborns born before the earthquake were excluded. Premature newborns and newborns with anomalies were also excluded, because they might have required special medical intervention and because there were no significant differences in the preterm birth or fetal malformation rates between the areas (Fujimori et al. 2014). Women who were pregnant at the time that they received the maternal survey questionnaire were also excluded from the analysis.

### Maternal and newborn characteristics

Before the main analysis, background information on the mothers and newborns in the two areas was compared. Maternal background information included age at the time of childbirth, delivery history (primiparous or multiparous), mode of delivery (vaginal or cesarean), depression status, and whether medical facilities had changed after the earthquake. Depression status was assessed by using Whooley’s 2-item case-finding instrument for depression, which assesses depressed mood and anhedonia in the preceding month (Whooley et al. 1997). Background information on newborns included gestational length, birth mass, thoracic circumference (TC) at birth, head circumference (HC) at birth, sex, and the proportion of newborns with birth mass under 2500 g.

### Study outcomes

#### Feeding methods and assessment of growth

Before assessing newborn growth status 1 month after birth, the exact timing of the 1-month postpartum examinations was compared. Thereafter, the average daily increases in mass (g/day), TC (mm/day), and HC (mm/day) were assessed. Average daily increases were estimated by subtracting the measurement at birth from the measurement taken at the 1-month postpartum examination and dividing the result by the number of days between the two measurements.

In this survey, mothers were asked what infant feeding methods were provided for their infants before the start of complementary foods (“Until now, what was the method of nutrition provided to your child until baby food began?”). Responses were categorized as exclusive breastfeeding (receiving breast milk only with no additional fluids unless mineral supplements or vitamins), exclusive formula feeding (receiving formula with no breast milk), and mixed breastfeeding (a combination of breastfeeding and infant formula). In order to assess the changes in the rates of feeding methods over time, the dates of birth were divided into four periods: March 11 to June 10, 2011; June 11 to September 10, 2011; September 11 to December 10, 2011; and December 11, 2011 to April 4, 2012. The feeding methods (exclusive breastfeeding, exclusive formula feeding, or mixed breastfeeding) of the infants born in each period were compared between the two areas.

#### Statistical methods

In order to examine the associated factors for newborn growth, a multivariate linear regression analysis was performed in each area, using the average mass gain per day (g/day) from birth to the first (1-month) postpartum examination as the outcome (dependent variable). The multivariate regression included the following independent variables: maternal age (years), maternal depression status (present or absent), change in medical facility after the disaster (yes or no), newborn sex, gestational age, feeding methods (exclusive breastfeeding or exclusive formula feeding), delivery (vaginal or cesarean section), and parity (primipara or multipara). Among these, maternal depression status and change in medical facility were considered disaster-related factors. SPSS version 21 (IBM Corp., Armonk, NY, USA) was used for the statistical analyses. Mann–Whitney *U* tests were conducted to compare continuous variables, and Chi-square tests were conducted to compare categorical variables. The extended Mantel–Haenszel Chi-squared test for a linear trend was used to analyze the trends in proportions. The level of statistical significance was set at *p* < 0.05.

## Results

### Enrollment

**Fig. 2:**
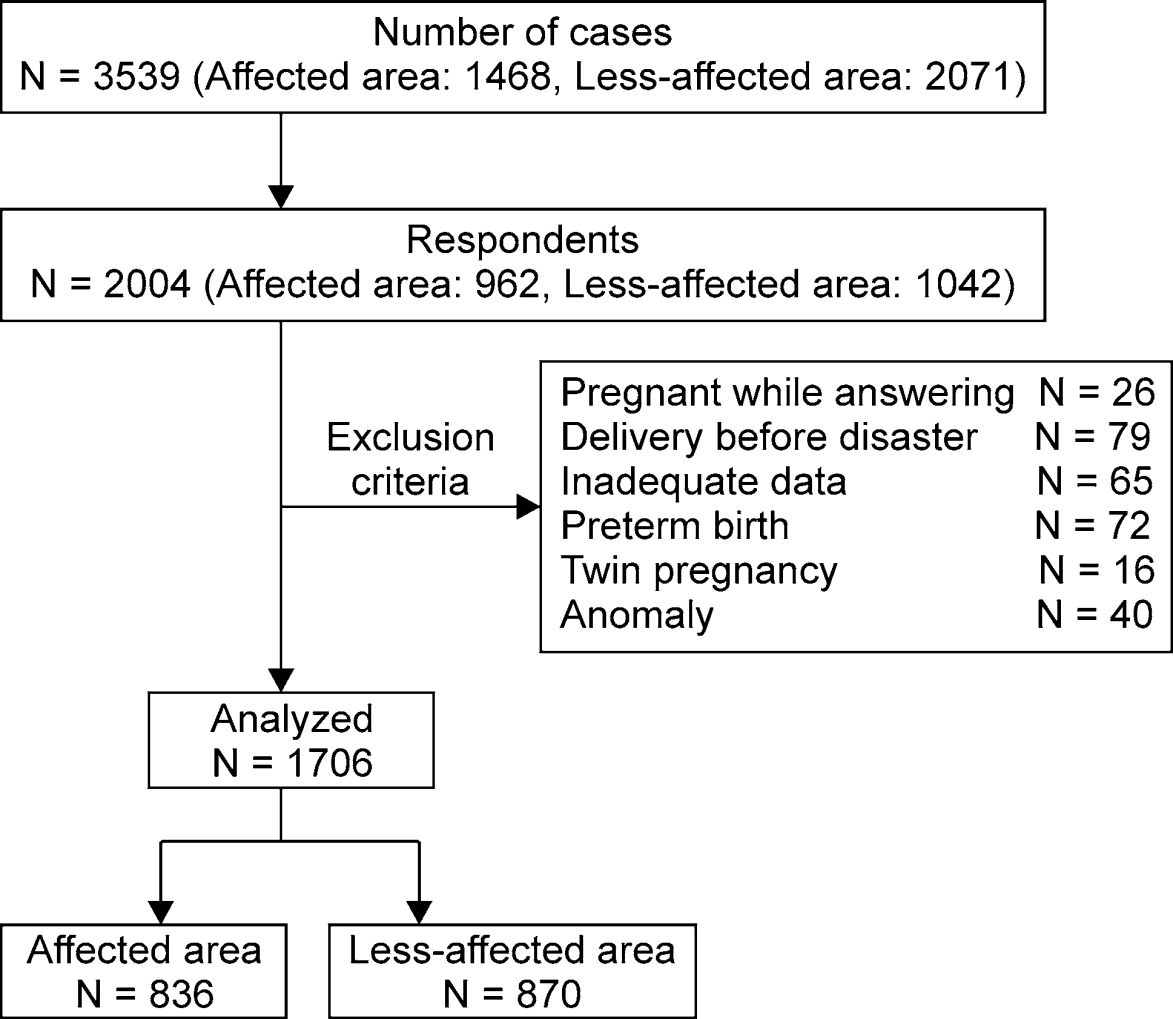
Study enrollment flowchart

The survey questionnaire was sent via mail to 1468 pregnant women in the affected area and 2071 women in the less-affected area. A total of 962 women (response rate, 65.5 %) and 1042 women (response rate, 50.3 %) responded to the questionnaire in the affected and less-affected areas, respectively. Based on the exclusion criteria, women who were pregnant at the time of the survey (*n* = 26), who had given birth before March 11, 2011 (*n* = 79), for whom insufficient data were available (*n* = 65), who had twins (*n* = 16), who had a newborn with an anomaly (*n* = 40), or who had a premature newborn born before the 37th week of pregnancy (*n* = 72) were excluded from the study. After applying these exclusion criteria, 836 and 870 women and their newborns were included in the analyses of the affected and less-affected regions, respectively (Fig. 2).

### Maternal characteristics

Although mean age at the time of delivery was significantly higher in the less-affected area (29.4 vs. 30.4; *p* < 0.01), there were no significant differences in the proportions of primiparous women (affected vs. less affected, 27.8 vs. 25.7 %; *p* = 0.42) or vaginal deliveries (affected vs. less affected, 82.4 vs. 80.4 %; *p* = 0.23). The proportion of women who had to change medical facilities after the disaster was significantly higher in the affected area (73.6 vs. 10.1 %; *p* < 0.01). Further, the proportion of women in a depressive state was significantly higher in the affected area (34.0 vs. 21.3 %; *p* < 0.01) (Table 1).

**Table 1:** Basic characteristics of mothers in the affected and less-affected areas; *n*—number of individuals included in the analyses

	Affected area	Less-affected area	*p* value
Age (years, mean ± SD)	29.4 ± 5.2	30.4 ± 5.0	<0.01
*n*	836	870	
Primipara [*n* (%)]	222 (27.8)	215 (25.7)	0.42
Multipara [*n* (%)]	577 (72.2)	621 (74.3)	
Total deliveries (*n*)	812	854	0.23
Vaginal delivery [*n* (%)]	669 (82.4)	684 (80.1)	
Maternal depressive state (*n*/total responses)	279/820	184/862	<0.01
Change in medical facility (*n*/total responses)	610/829	87/864	<0.01

### Newborn characteristics

No significant differences were observed between the two areas in terms of the mean gestational age at delivery (affected vs. less affected, 39.1 vs. 39.0 weeks; *p* = 0.17), the proportion of low birth-mass newborns (affected vs. less affected, 5.1 vs. 6.3 %; *p* = 0.31), or the proportion of male newborns (affected vs. less affected, 51.4 vs. 49.2 %; *p* = 0.38). Further, there were no significant differences in mean birth mass (affected vs. less affected, 3067 g vs. 3055 g; *p* = 0.47), TC (affected vs. less affected, 32.0 cm vs. 32.0 cm; *p* = 0.85), or HC (affected vs. less affected, 33.2 cm vs. 33.2 cm; *p* = 0.93) (Table 2).

**Table 2:** Birth outcomes in the affected and less-affected areas

	Affected area	Less-affected area	*p* value
Gestational age (weeks, mean ± SD) (*n*)	39.1 ± 1.1 (832)	39.0 ± 1.1 (867)	0.17
Birth mass (g, mean ± SD) (*n*)	3067.4 ± 351.4 (823)	3055.5 ± 369.4 (864)	0.47
Birth thoracic circumference (cm, mean ± SD) (*n*)	32.0 ± 1.5 (817)	32.0 ± 1.6 (855)	0.85
Birth head circumference (cm, mean ± SD) (*n*)	33.2 ± 1.3 (815)	33.2 ± 1.4 (852)	0.93
Total number of newborns (*n*)	823	865	
Male [*n* (%)]	423 (51.4)	426 (49.2)	0.38
Female [*n* (%)]	400 (48.6)	439 (50.8)	
Proportion of low birth weight newborns	823	864	0.31
*n* (%)	42 (5.1)	54 (6.3)	

### Growth at the first postpartum examination

The average interval between birth and the postpartum examination was 33.2 days and 32.8 days in the affected and less-affected areas, respectively, which were not significantly different (*p* = 0.66). There were also no significant differences between the two regions in terms of average mass gain per day, average TC gain per day, or average HC gain per day at the first postpartum examination (Table 3).

**Table 3:** Results of growth at the first postpartum examination

	Affected area	Less-affected area	*p* value
Timing of 1-month postpartum examination (days, mean ± SD) (*n*)	33.2 ± 8.8 (812)	32.8 ± 5.8 (841)	0.66
Mass gain per day (g/day, mean ± SD)	35.9 ± 10.4 (753)	35.9 ± 10.1 (761)	0.88
TC gain per day (mm/day, mean ± SD)	1.29 ± 0.05 (719)	1.30 ± 0.05 (755)	0.75
HC gain per day (mm/day, mean ± SD)	1.08 ± 0.04 (725)	1.07 ± 0.04 (754)	0.52

*TC* thoracic circumference, *HC* head circumference

### Feeding methods

There were no significant differences between the affected and less-affected areas in terms of the newborns’ feeding methods during the first and second of the four periods examined. However, feeding methods differed significantly between the areas during the third and fourth periods (*p* < 0.05 and *p* < 0.01, respectively). When each method was compared, no significant change in the rate of exclusive breastfeeding could be observed across time periods in the affected or less-affected area (*p* = 0.17 and *p* = 0.38, respectively). With respect to trends in the rates of exclusive formula and mixed breastfeeding, no statistically significant change was seen in the less-affected area (*p* = 0.37 and *p* = 0.62, respectively), but significant differences were found in the affected area (*p* < 0.05 and *p* < 0.01, respectively) (Table 4).

**Table 4:** Period of delivery and feeding methods in the affected and less-affected area

Period of delivery	Affected area				Less-affected area				*p* value^a^
	Total	Feeding methods			Total	Feeding methods			
		Exclusive breastfeeding	Mixed breastfeeding	Exclusive formula feeding		Exclusive breastfeeding	Mixed breastfeeding	Exclusive formula feeding	
2011.03.11–2011.06.10 *n* (%)	240	54 (22.5)	167 (69.6)	19 (7.9)	206	59 (28.6)	135 (65.5)	12 (5.8)	0.27
2011.06.11–2011.09.10 *n* (%)	268	81 (30.2)	160 (59.7)	27 (10.1)	266	74 (27.8)	175 (65.8)	17 (6.4)	0.18
2011.09.11–2011.12.10 *n* (%)	208	57 (27.4)	124 (59.6)	27 (13.0)	255	70 (27.5)	170 (66.7)	15 (5.9)	<0.05
2011.12.11–2012.04.04 *n* (%)	116	35 (30.2)	63 (54.3)	18 (15.5)	142	49 (34.5)	88 (62.0)	5 (3.5)	<0.01
*p* value for trend^b^		0.17	<0.01	<0.05		0.38	0.62	0.37	

^a^Chi-square test was used to compare affected and less-affected areas

^b^Extended Mantel–Haenszel–Chi-square test for a linear trend was used to analyze the trend of breastfeeding and exclusive formula-feeding ratios

### Factors influencing newborn growth at the first postpartum examination

The multivariate regression analyses showed that male sex of the newborn and exclusive breastfeeding were significantly and independently associated with average mass gain per day at the first postpartum examination (the outcome variable) in both areas (*p* < 0.01 for each assessment). In addition, vaginal delivery was a significant independent factor (*p* < 0.01) in the affected area; maternal age (*p* < 0.01) and parity (*p* < 0.01) were significant independent factors in the less-affected area. Maternal depressive state and change in medical facility (impact of the disaster) were not significant independent factors in either area (Table 5).

**Table 5:** Factors related to newborn growth at first postpartum examination: multiple linear regression analysis; *B* partial regression coefficient; *p* *p* value

Predictor	Affected area		Less-affected area	
	B	*p*	B	*p*
Maternal age	−0.08	0.28	−0.27	<0.01
Depression state	1.25	0.11	−1.65	0.06
Change in medical facility	0.43	0.58	0.85	0.58
Sex	−5.04	<0.01	−5.80	<0.01
Gestational age	−0.20	0.57	−0.22	0.52
Feeding method	−3.16	<0.01	−3.48	<0.01
Vaginal delivery	−2.74	<0.01	−1.50	0.11
Parity	−0.10	0.91	2.12	<0.01

*Outcome* mass gain per day (g/day)

*Independent variables* maternal age, depression state (1, no; 2, yes), changed facility after disaster (1, no; 2, yes), sex (1, male; 2, female), gestational age, feeding methods (1, breast milk only; 2, used formula), vaginal delivery (1, vaginal delivery; 2, cesarean section), parity (1, primipara; 2, multipara)

## Discussion

The present analyses of prefectural survey data indicated that there were no significant differences between the affected and less-affected areas in birth outcomes, rates of exclusive breastfeeding, or growth at the first postpartum examination, despite the obvious differences in maternal environments. Further, earthquake-related factors, such as maternal depressive state and change in medical facility, which were different between the affected and less-affected, were not independently associated with growth at the 1-month first postpartum examination.

It was found here that pregnant women in the affected area showed signs of significant mental health deterioration after the earthquake. This is in line with previous findings that were obtained by using the same screening tool in Fukushima City, which showed that mothers in areas most affected by the nuclear accident, i.e., those with higher radiation levels, were more likely to have depressive symptoms (Goto et al. 2014; 2015).

Regarding pregnancy outcomes, several reports have suggested that the earthquake disaster affected fetal outcomes (Camacho 2008; Chang et al. 2002; Catalano et al. 2006; Engel et al. 2005; Eskenazi et al. 2007; Fukuda et al. 1998; Saadat 2008; Smits et al. 2006), while others found no associations (Berkowitz et al. 2003; Hamilton et al. 2009; Leaderman et al. 2004). The present study excluded cases needing special medical intervention and showed that mental and physical stress caused by the earthquake did not affect newborns’ anthropometric measures at birth. In earthquakes, other natural disasters, and forced evacuations, children are subjected to harmful situations, such as poor nutrition, and it is widely agreed that prompt political and humanitarian support are important (Assefa et al. 2001; Salama et al. 2001; Sun et al. 2013). In the present analysis of factors associated with newborns’ average mass gain per day at the first (1-month) postpartum examination, disaster-related items (change in medical facilities and depressive state) did not affect mass gain. With respect to the rates of exclusive breastfeeding, no changes were evident among the four investigated time periods. These data suggest that the majority of postpartum women in the affected area may have had appropriate and accurate support and guidance regarding feeding practices in an emergency. This fits with the introduction of education programs and public campaigns that have followed previous earthquakes in Japan, for example, the Chuetsu earthquake in 2004, where mothers were advised on the importance of breastfeeding after natural disasters (Joliffe 2005). It has been noted that efficient systems have been established for rescue work following disasters in Japan, wherever powerful earthquakes have occurred in the past (Zaré and Afrouz 2012). These systems have likely resulted from guidelines from the Japan Society of Obstetrics and Gynecology (JSOG) and the Japan Association of Obstetricians and Gynecologists (JAOG) to provide a better understanding for pregnant women and prompt political and humanitarian support after the earthquake (JSOG and JAOG 2014). In addition, in Japan, the Ministry of Health, Labour and Welfare required administrative dieticians to support specific food service facilities and develop a food supply system for times of emergency (Ministry of Health, Labour and Welfare 2008).

In contrast, there was a significant increase in the rate of exclusive formula feeding across time periods in the affected area, indicating that mothers may have chosen exclusive formula feeding because they feared contamination from radioactive substances that may be transmissible through breast milk (Unno et al. 2012). Of note, the number of exclusively formula-feeding mothers was small, and the obtained result needs to be interpreted with caution. We were unable to determine the levels of support provided in different areas; however, the observed increase in the rate of exclusive formula feeding across time periods may be an indication that support and instruction did not reach all mothers in the most affected areas that had higher radiation levels or that distribution of infant formula was poorly targeted. Future studies examining whether rates of exclusive breastfeeding differ according to levels of emergency support in disaster areas would help to shed light on the relationship between the two. The continued monitoring of radiation doses and mothers’ feeding behavior, along with the promotion of public understanding of the transmission of radioactive substances through breast milk, remains challenging. Detrimental damage to basic infrastructures, including sewage, water, housing, and health care indicates that post-emergency circumstances require continuous and improved guidance on infant feeding practices to prevent early cessation of exclusive breastfeeding and replacement with formula.

The Great East Japan Earthquake, the resulting tsunami, and the subsequent Fukushima Daiichi nuclear crisis represented an unprecedented and complex sequence of disasters. In response, the Fukushima Health Management Survey was sponsored by the government to examine long-term health conditions of Fukushima residents and to investigate the long-term health effects of low-dose radiation (Yasumura et al. 2012). In Japan, few maternal surveys have involved all pregnant women in the community, and the results of the present large-scale study, led by the government, are considered extremely valuable.

The present study includes a number of methodological limitations that need to be considered. First, this was a descriptive study based on a relatively small sample size which did not allow establishment of cause and effect. Second, the study relied only on data that were collected by using a questionnaire. However, the Fukushima Health Management Survey has been used as the basis for a number of studies (Yasumura et al. 2012) and has received support for ongoing monitoring of health data from the Japanese government. In addition, all mothers in Japan have Maternal and Child Health Handbooks, and the mothers in the survey used here were asked to refer to these when completing the questionnaire, which helped to reduce recall bias and improve accuracy, so their answers were considered quite accurate. Third, because the response rate was only approximately 60 %, the data may have overestimated the actual incidence of negative outcomes if there was an overrepresentation of women who had been affected most by the disasters. Fourth, it was impossible to ascertain the reasons behind infant feeding method by mothers or establish their awareness of good feeding practices during emergencies—a qualitative study would help improve our understanding of these issues. Fifth, infant growth development was based on a short time interval of 1 month. Sixth, infant feeding practices could only be assessed at a single time point and to compare with those prior to the earthquake was not possible. Further, radiation contamination between areas could not be compared. Finally, this survey targeted mothers of infants born within a year of the earthquake, and the residents of Soso District, who were forced into long-term evacuations due to the complex disaster. These mothers are expected to continue to experience stress in the future. Thus, a more comprehensive investigation of the influence of this disaster on pregnancy and infant care will follow, but worldwide interest warrants prompt reporting of what we learn as we learn it.
